# Cohort analysis of 50% lethal area (LA50) and associating factors in burn patients based on quality improvements and health policies

**DOI:** 10.1038/s41598-023-45884-9

**Published:** 2023-11-03

**Authors:** Reza Shahriarirad, Ramin Shekouhi, Sara Sadat Nabavizadeh, Mitra Zardosht, Seyed Mohammad Kazem Tadayon, Meysam Ahmadi, Abdolkhalegh Keshavarzi

**Affiliations:** 1 Thoracic and Vascular Surgery Research Center, Shiraz University of Medical Science, Shiraz, Iran; 2 Burn and Wound Healing Research Center, Plastic and Reconstructive Surgery Ward, Shiraz University of Medical Science, Shiraz, 71348-14336 Iran; 3 Department of Surgery, Shiraz University of Medical Sciences, Shiraz, Iran; 4 Otolaryngology Research Center, Shiraz University of Medical Sciences, Shiraz, Iran; 5 Colorectal Research Center, Shiraz University of Medical Sciences, Shiraz, Iran; 6 School of Medicine, Shiraz University of Medical Science, Shiraz, Iran

**Keywords:** Epidemiology, Trauma, Risk factors, Epidemiology, Outcomes research

## Abstract

Burn injuries are among the common traumatic injuries, which can be accompanied with lifelong morbidity and mortality. The Lethal Area Fifty Percent (LA50) index is another reliable outcome measurement tool that assesses the standard of medical care at burn centers. It is widely used as a benchmark for assessing the quality of burn care and is considered the percentage at which 50% of burn patients are expected to die because of burn-related injuries. We aimed to determine and compare the LA50 in burn patients admitted to Shiraz Burn Referral Centers in 2018–2021 and 2011–2018 with regard to improving the quality of special care and infection control in the new hospital. We conducted a retrospective cohort analysis on patients admitted to Amir al-Momenin Burn Injury Hospital in Shiraz, Fars, Southern Iran. Data were retrospectively gathered from March 2011 to January 2022, and subsequently analyzed with standard statistical analysis, and also multivariate and probability analysis. A total of 7382 patients with acute burns injuries were identified. Among them, 4852 (65.7%) patients were men, and the median age was 27 years [Q1–Q3 7–40; range 1–98]. Most of the patients were in the pediatric and early adulthood age range, with 76.2% being younger than 40 years old. The median TBSA was 24% [IQR 14, 43], and the median duration of hospitalization was 11 [IQR 11] days. Most injuries were secondary to flame and fire (33.5%; n = 2472). The mortality rate in our study was 19.0% (n = 1403). We evaluated our patients based on two main time intervals: March 2011 till February 2018 (n = 3409; 46.2%), and March 2018 to January 2022 (n = 3973; 53.8%). Based on multivariate analysis, the second interval of our study was significantly correlated with a more female patients, higher age, lower TBSA, less burn injuries due to scald, contact, but more frequent fire and flame injuries, and also lower mortality rate. Factors correlated with higher mortality included male gender, older age, shorter hospitalization duration, higher TBSA, etiology of fire and flame, and accidental burn injuries. A Baux score of 76.5 had a sensitivity of 81.1%, specificity of 87.3%, accuracy of 86.1% in predicting mortality among our patients. The mortality probability for the study intervals were 20.67% (SD 33.0%) for 2011–2018, and 17.02% (SD 29.9%) for 2018–2022 (*P *< 0.001). The LA50 was 52.15 ± 2 for all patients. This ammount was 50 ± 2% in 2011–2018, and 54 ± 2 in 2018–2022 (*P *< 0.001). The mean LA50 values showed significant improvements following significant modifications in our critical care for burn victims, including augmented intensive care unit capacity, prompt relocation of inhalation burn cases to the intensive care unit, establishing a well-trained multidisciplinary team, and improved infection control. To improve outcomes for burn patients in developing countries, major changes should be made in the management of burn patients and LA50 is a reliable assessment tool for evaluating the how these changes affect patient’s outcomes.

## Introduction

Burn injuries are considered a common cause of trauma that significantly affects patients’ life, resulting in high morbidity and mortality, particularly in developing countries^1–6^. The classification of burns typically relies on their underlying cause, with thermal, electrical, and chemical injuries being the most commonly recognized categories^7^. The optimal management of burn patients has been a subject of significant debate, which can be costly, requiring prolonged hospitalization that can potentially lead to life-long sequelae^7,8^. It requires a comprehensive and interdisciplinary approach from the initial critical stage to recovery and rehabilitation^9^.

Recent developments in burn management, including optimal tissue excision and grafting, enhanced infection prevention and control measures, specialized critical care interventions, as well as tailored nutritional interventions have collectively contributed to the reduction of mortality rates in burn patients^10,11^. To assess the outcomes of burn patients, multiple factors must be considered including survival rate, wound healing, functional status, burn-related complications, and overall mortality rate. The Lethal Area Fifty Percent (LA50) index is another reliable outcome measurement tool that assesses the standard of medical care at burn centers. It is widely used as a benchmark for assessing the quality of burn care and is considered the percentage at which 50% of burn patients are expected to die because of burn-related injuries^12,13^. Repeated measurements of this index can be used to determine the effectiveness of burn care to patients.

The estimated LA50 in the United States at its first description in 1940s was nearly 40%^14^. Nevertheless, significant improvements in burn and critical care management through the past decades led to a current LA50 value of nearly 90%^15^, meaning that patients now have a greater likelihood of surviving extensive burn injuries. In the developing countries however, the mortality rates are still high and there is not enough evidence about their LA50 values. After to 2018, our burn center witnessed significant modifications in the critical care for burn victims, including augmented intensive care unit capacity, prompt relocation of inhalation burn cases to the intensive care unit, and the integration of high-efficiency particulate air filters for the burn units. Subsequently, this study was designed to compare the outcomes of our patients prior and after this modification. Moreover, the study aims to characterize the changes in LA50 subsequent to these advancements in burn care.

## Materials and methods

### Study design and setting

We conducted a retrospective cohort analysis of patients admitted to Amir al-Momenin Burn Injury Hospital in Shiraz, a 75-bed tertiary care hospital that serves as a burn and plastic surgery referral center for over five million people in Fars province. Data were retrospectively gathered from March 2011 to January 2022, and patients were grouped throughout two consecutive time periods (2011–2018 and 2018–2022).

The database registry was initiated by Burn and Wound Healing Research Center located in Amir-al Momenin Hospital. The data element for this database questionnaire was designed by clinicians and epidemiologists. Dedicated clinical research assistants collected all data using a standardized electronic case report form. To remove mistakes caused by missing or inconsistent data, an extra researcher verified the correctness of data entry for 15% of patients. The STROBE (Strengthening the Reporting of Observational Studies in Epidemiology) guidelines of cohort studies was used for reporting and writing this study.

### Study population

All patients admitted to Amir al-Momenin Burn Injury Hospital for acute burn injuries between March 2011 and January 2022 were included in our study. To focus our study on patients with burn injuries and receiving related in-hospital treatment, we excluded cases with concurrent additional serious injuries and traumas who were initially admitted to a primary trauma center, such as head trauma, major torso trauma, or long bone fractures, or those who died in the emergency department (dead-on-arrival) or were admitted for less than 24 h and did not have the opportunity to receive medical services, or without a comprehensive registry of medical records. Based on our hospital policy, all patients were evaluated during the first hour of admission and transferred to either the appropriate ward or the intensive care unit (ICU).

### Data sources

Data were obtained from the Health Information System (HIS)—a population-based administrative database that tracks all care hospitalizations in the province of Fars, and also patient medical records. Collected data included demographical features such as age, gender, and admission year, and also clinical features such as etiology of burn injuries (defined by Herndon et al.^6,16^ and also previous studies, including flame burn, scald burn, contact burn, electrical burn, chemical burn, and explosion/blast burn), the reason of burn injuries (accident, suicide or homicide), total body surface area (% TBSA) burnt, length of hospitalization, and patient outcomes (mortality, transfer, discharge, and left against medical advice).

### Ethical considerations

The ethical review committee of the Shiraz University of Medical Sciences (Ethical approval code: IR.SUMS.MED.REC.1400.638) approved the study. All experiments were performed in accordance with relevant guidelines and regulations. Based on the retrospective nature of the study, written informed consent waiver was approved by the Ethics committee of Shiraz University of Medical Sciences. Patients’ information were anonymized prior to analysis and confidentiality was assured by the researcher.

### Statistical analysis

Data were initially entered into SPSS version 26.0 (SPSS Inc., Chicago, Illinois, USA) and subsequently analyzed. Qualitative data were presented in frequency, and percentages (%). Based on the skewed distribution of quantitative variables such as length of hospitalization, TBSA, and age, these variables were reported using the median, quartiles [Q1–Q3] and interquartile range [IQR]. The Mann–Whitney U and Kruskal Wallis test was done to compare the medians of two categorical variables, while the Chi-square test was used to compare categorical data.

In addition, binary univariable logistic regression was utilized to quantify the influence of a probable underlying factor on mortality in patients with acute burns by classifying the patients' outcomes as mortality or survival. Then, multivariable logistic regression was performed to modify the influence of factors whose *P* values were less than 0.2 in univariable logistic regression. The odds ratio (OR) and its associated 95% confidence interval were reported. In addition, the lethal area fifty percentage (LA50) is defined as the TBSA of burn with a 50% probability of death in the patient and was calculated in our study using a Probit model. We also calculated the Baux score in our patients, which is the sum of TBSA and the patients age, and evaluated its correlation with mortality along with assigning a relative cut-off based on receiver operating characteristic (ROC) curve analysis^17^. All tests were two-tailed, and a *P* value of less than 0.05 was considered the threshold for statistical significance.

### Ethics approval and consent to participate

The present study was approved by the medical ethics committee of the academy. The permission was obtained from the medical ethics committee of Shiraz University of Medical Sciences. Based on the retrospective nature of our study, written informed consent was waived by the Ethics committee of Shiraz University of Medical Sciences, and their information was obtained from their hospital records. Permission to carry out the study and access patient records was sought from the Shiraz University of Medical Science administrators and the study was conducted in compliance in accordance with the relevant guidelines and regulations and the Declaration of Helsinki and was also approved by the ethics committee of the university.

## Results

**Figure 1:**
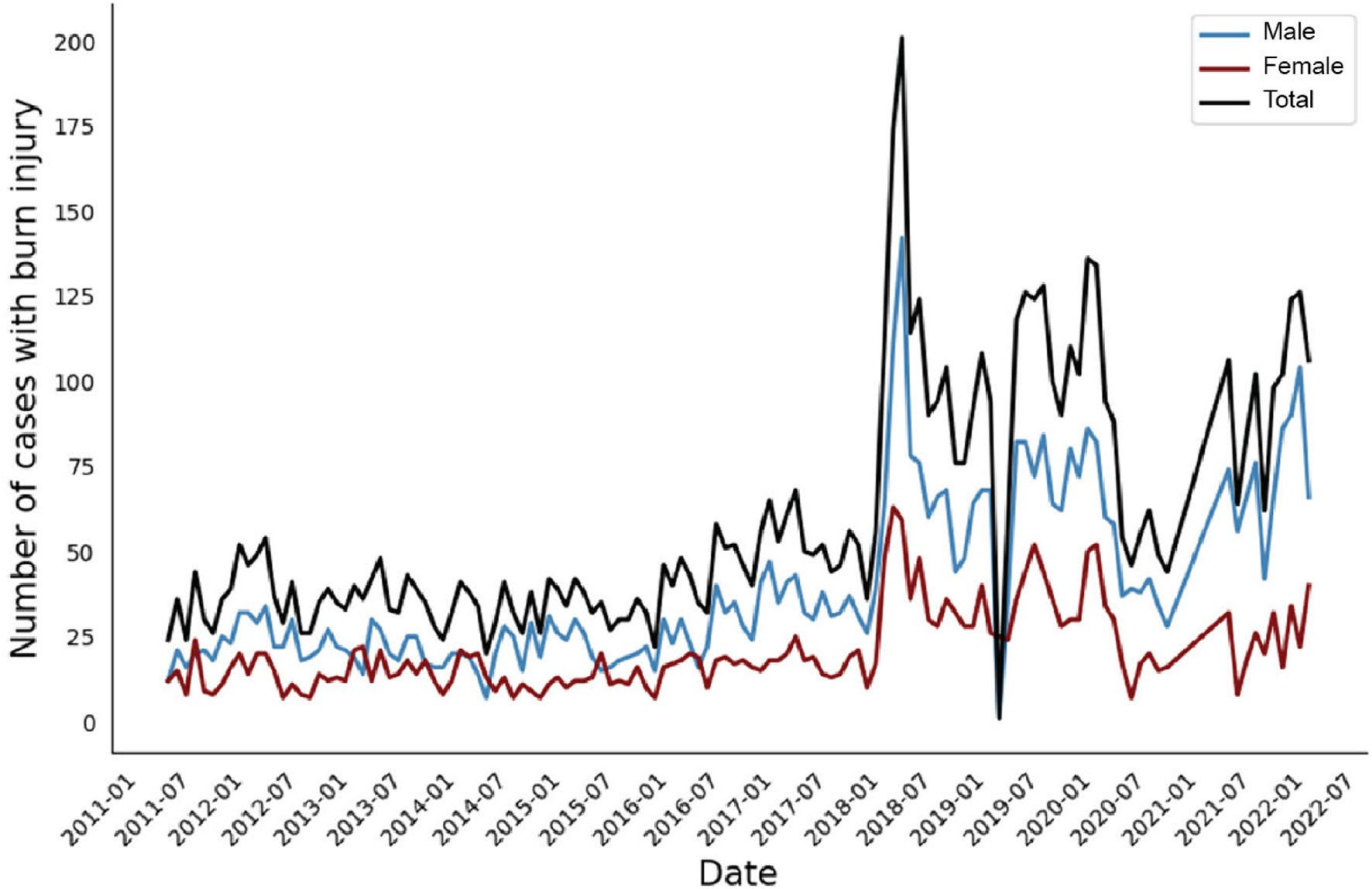
Frequency of hospitalizations per month due to burn injuries, along with gender distribution in a referral burn center in southern west Iran from 2011 to 2022.

During the study period (2011–2022), a total of 7382 patients with acute burns injuries who fulfilled the study’s inclusion criteria were identified. Among them, 4852 (65.7%) patients were men, and the median age was 27 years [Q1–Q3 7–40; range 1–98] (Fig. 1). The baseline features of the patient and injury in our study and a comparison between 2011–2018 and 2018–2022 are shown in Table 1. We evaluated our patients based on two main time intervals: March 2011 till February 2018, and March 2018 to January 2022. The first interval included 3409 (46.2%) of the patients, while the second interval consisted of 3973 (53.8%) patients, demonstrating an annual rate of 487 cases and 993 cases (2.04-fold increase).

**Table 1:** Comparison of the baseline characteristics of patients with acute burn injury admitted to Shiraz Burn Center between 2011–2018 and 2018–2022.

Variable	Total; N = 7382	Year		*P* value
		2011–2018; n = 3409	2018–2022; n = 3973	
Gender; n (%)				
Male	4852 (65.7)	2147 (63.0)	2705 (68.1)	< 0.001
Female	2530 (34.3)	1262 (37.0)	1268 (31.9)	
Age (years); median [Q1–Q3]	27 [7–40]	25 [7–36]	29 [8–42]	< 0.001
Age group; n (%)				
0–20 years	2790 (37.8)	1317 (38.6)	1473 (37.1)	< 0.001
21–40 years	2835 (38.4)	1412 (41.4)	1423 (35.8)	
41–60 years	1208 (16.4)	492 (14.4)	716 (18.0)	
> 60 years	549 (7.4)	188 (5.5)	361 (9.1)	
Burn etiology; n (%)				
Contact	232 (3.1)	42 (1.2)	190 (4.8)	< 0.001
Scald	1642 (22.2)	705 (20.7)	937 (23.6)	
Fire/flame	2472 (33.5)	1306 (38.3)	1166 (29.3)	
Electrical	357 (4.8)	156 (4.6)	201 (5.1)	
Chemical	92 (1.2)	34 (1.0)	58 (1.5)	
Explosion/blast	2023 (27.4)	918 (26.9)	1105 (27.8)	
Burn intention; n (%)				
Accidental	6643 (90.0)	2929 (85.9)	3714 (93.5)	< 0.001
Homicidal	102 (1.4)	52 (1.5)	50 (1.3)	
Suicidal	598 (8.1)	389 (11.4)	209 (5.3)	
TBSA (%); median [Q1–Q3]	21 [11–39]	24 [14–43]	18 [9–36]	< 0.001
TBSA group (%); n (%)				
0–10	1741 (23.8)	527 (15.5)	1214 (31.0)	< 0.001
11–20	1889 (25.8)	957 (28.1)	932 (23.8)	
21–30	1202 (16.4)	627 (18.4)	575 (14.7)	
31–40	767 (10.5)	372 (10.9)	395 (10.1)	
41–50	499 (6.8)	269 (7.9)	230 (5.9)	
51–60	373 (5.1)	174 (5.1)	199 (5.1)	
61–70	221 (3.0)	115 (3.4)	106 (2.7)	
71–80	197 (2.7)	98 (2.9)	99 (5.1)	
81–90	198 (2.7)	111 (3.3)	87 (2.2)	
91–100	241 (3.3)	157 (4.6)	84 (2.1)	
Length of hospitalization (days); median [Q1–Q3]	11 [7–18]	12 [7–19]	11 [7–17]	< 0.001
Baux score	53 [29–78]	54 [30–80]	52 [28–75]	< 0.001
Mortality rate; n (%)	1403 (20.7)	734 (23.6)	669 (18.3)	< 0.001

*P* values less than 0.05 was considered significant.

*IQR* Interquartile range, *TBSA* Total burn surface area.

**Figure 2:**
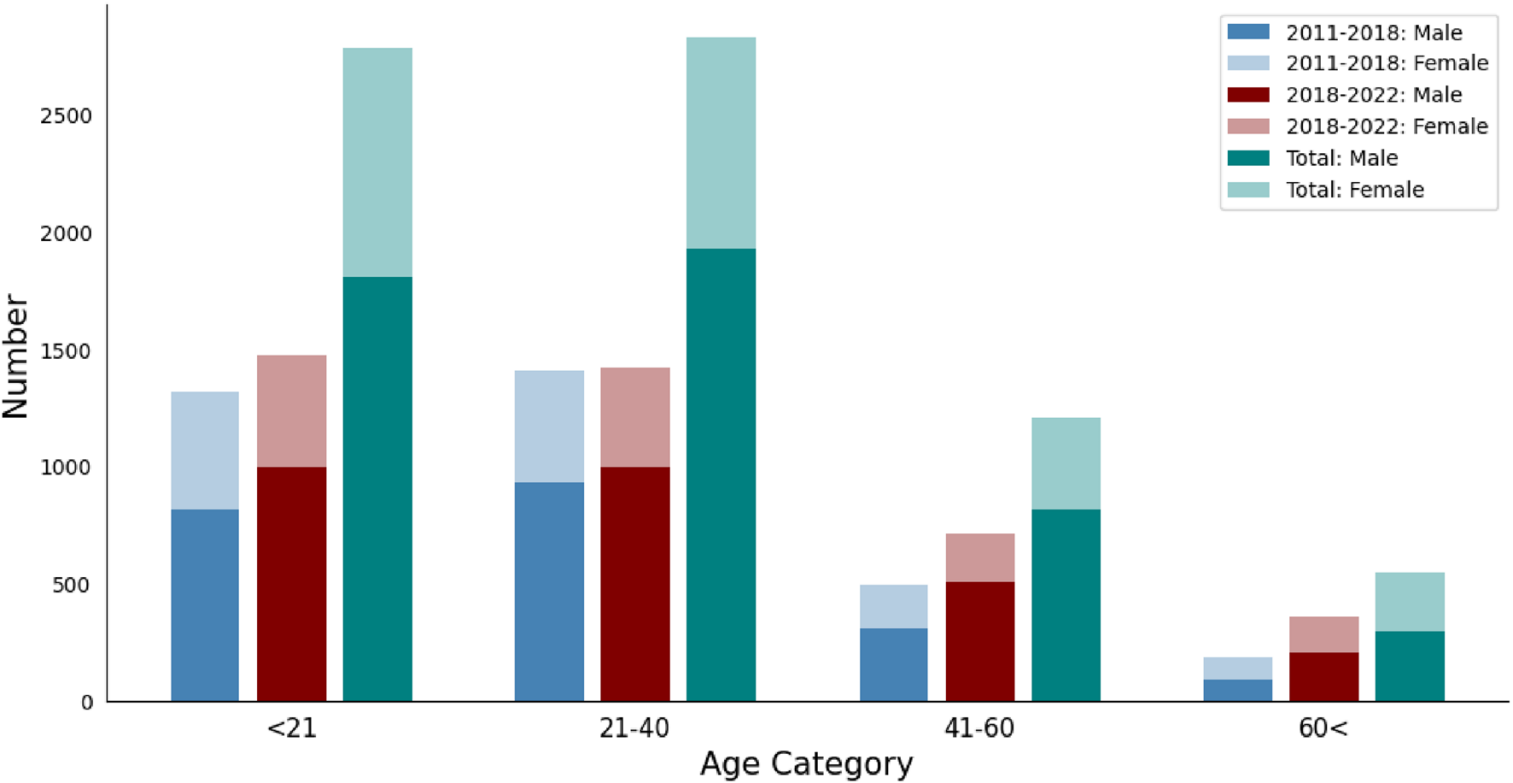
Frequency of hospitalized burn injuries in each age category by gender in a referral burn center in southern west Iran from 2011 to 2022, and comparison between 2011–2018 and 2018–2022.

Most of the patients were in the pediatric and early adulthood age range, with 76.2% being younger than 40 years old. In addition, patients were significantly younger during the first interval of our study period (*P *< 0.001) and the age distribution shifted towards older ages during the last four years, especially among the 41– 60 and above 60 age group (from 14.4 to 18.0% and 5.5 to 9.1%, respectively) (Fig. 2). The median age was similar across the sexes (*P *= 0.509). However, the incidence of burn injuries among male patients significantly increased during our timeframe, in which the proportion of male patients increased from 63% in 2011–2018 to 68% in the 2018–2022 (OR = 0.797, CI 95% 0.724–0.878; *P *< 0.001).

The median TBSA throughout our study was 21% [IQR 28], which was significantly higher in females than males (24% [IQR 33] and 20% [IQR 26], respectively; *P *< 0.001). The median TBSA was 16%, 27%, 23% and 17% for under 21, 21–40, 41–60 and above 60 age group, respectively. Furthermore, there was a considerable drop in TBSA in 2018–2022 compared to 2011–2018 (18% [IQR 27] vs. 24% [IQR 29], respectively; *P *< 0.001).

We had 80 patients with TBSA of 100%, which all died after a median of 3 days (range 2–17), with 29 (37.2%) of them during the initial 48, and 16 (20.5%) during the initial 72 h.

**Figure 3:**
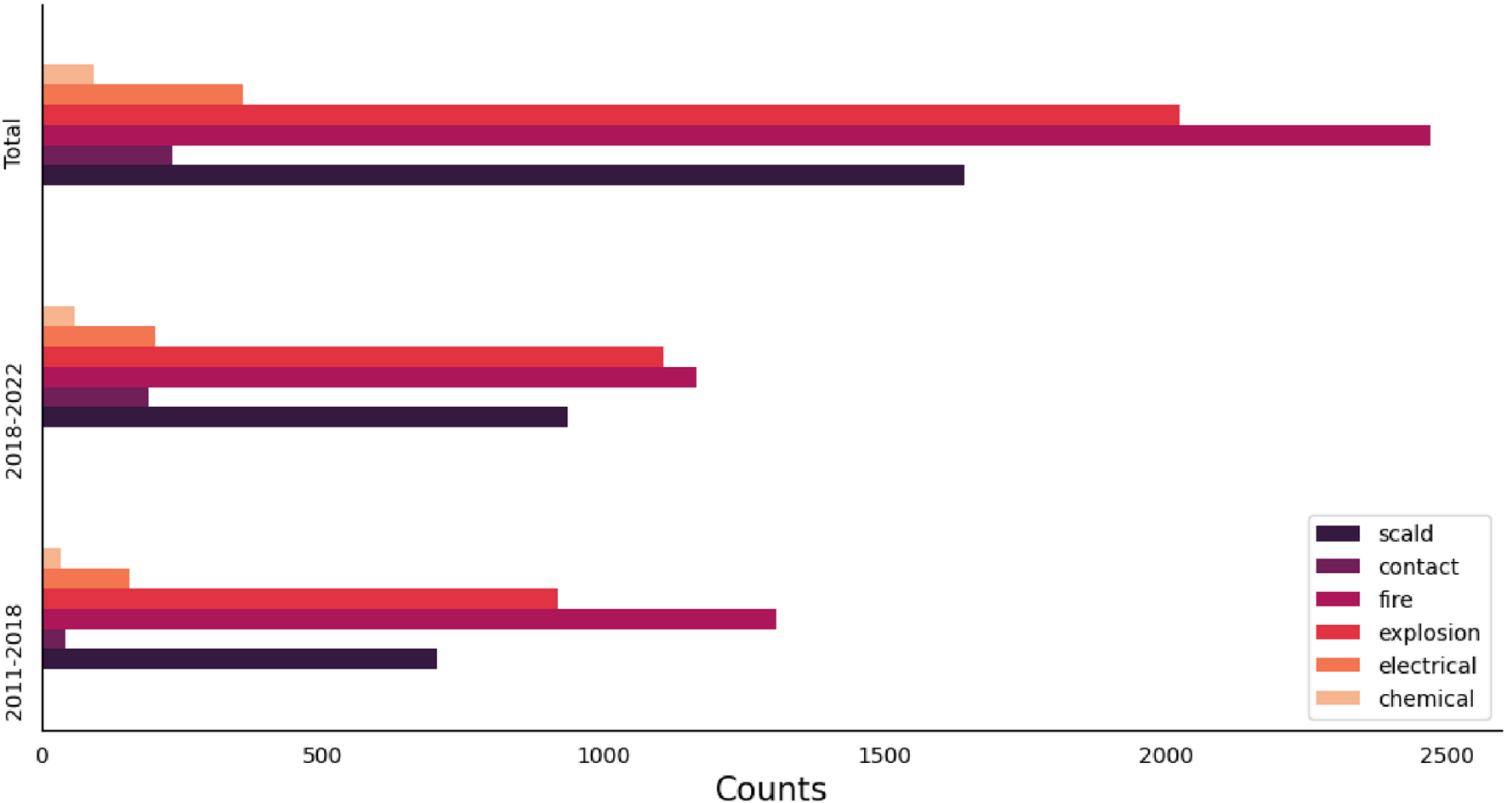
Frequency of hospitalized burn injuries along with burn etiology distribution in a referral burn center in southern west Iran from 2011 to 2022, and comparison between 2011–2018 and 2018–2022.

Most injuries were secondary to flame and fire (33.5%; n = 2472), followed by an explosion/blast (27.4%; n = 2023) and scald (22.2%; n = 1642) burns, while a small proportion (1.2%; n = 92) were chemical in nature (Fig. 3). There was a significant increase in causes of burn due to scald (from 20.7% to 23.6%; OR 1.184; CI 95% 1.060–1.322; *P *= 0.003) and objects (1.2% to 4.8%; OR 4.026; CI 95% 2.873–5.642; *P *< 0.001), but a significant decrease in causes of burn due to fire (38.4–29.4%; OR 0.667; CI 95% 0.605–0.735; *P *< 0.001).

On the other hand, accidental burns grew with time (from 86.9% in 2011–2018 to 93.5% in 2018–2022; OR 2.159; CI 95% 1.838–2.536), but suicide burns dropped over time (from 11.5% in 2011–2018 to 5.3% in 2018–2022; OR 0.426; CI 95% 0.357–0.507; *P *< 0.001), however, homicidal changes were not significant (1.5% vs. 1.3% for 2011–2018 and 2018–2022, respectively; *P *= 0.299). Causes of suicide were documented in the recent years (2018–2022), in which the most common cause was family issues (71.29%), followed by mental health issues (20.57%), and financial issues (2.39%).

The total median duration of hospitalization was 11 [IQR 11] days, with no significant difference based on gender (*P *= 0.118). However, In the latter four years of the research, hospital stays were considerably shorter than in the first four (11 [IQR 10] vs. 12 [IQR 12], respectively; *P *< 0.001). Also, there was a significant decrease in hospital stay as the date of the year increases (Correlation Coefficient − 0.038; *P *= 0.001), and a significant increase in duration with the increase of age (Correlation Coefficient 0.100; *P *< 0.001).

Regarding patient’s outcomes, 19.0% (n = 1403) died, 72.7% (n = 5352) were discharged, 0.4% (n = 30) were transferred to other hospitals, 7.6% (n = 558) left against medical advice. Meanwhile, the mortality rate was significantly higher among male patients (male/female 52.6% vs. 47.4%; OR 1.988; CI 95% 1.766–2.237; *P *< 0.001). We achieved a significant decrease in mortality rates from the first interval to the second interval of our study period, in which the mortality rate in 2011–2018 was 21.5%, while in 2018–2022 was 16.9% (OR 0.741; CI 95% 0.659–0.832; *P *< 0.001). When evaluating the changes in mortality patterns among gender, there was a significant decrease in female mortality rates throughout the two study intervals (30.6% vs. 22.1% for 2011–2018 and 2018–2022, respectively; OR 0.642; CI 95% 0.537–0.768; *P *< 0.001); however, this change was not significant among male patients (16.2% vs. 14.5% for first and second interval, respectively; *P *= 0.093). Furthermore, the pattern of decrease in mortality was significant among all age groups (≤ 20: OR 0.660, *P *= 0.001; 21–40: OR 0.765, *P *= 0.003; 41–60: OR 0.686, *P *= 0.006; > 60: OR *P *= 0.004).

We evaluated our data based on univariate and multivariate analysis, to access factors correlated with burn patient mortality. As demonstrated in Table 2, the second interval of our study (2018–2022) was significantly correlated with a lower mortality rate compared to the first interval (2011–2018). Further risk factors for mortality included male gender, older age, shorter hospitalization duration, higher TBSA, etiology of fire and flame, and accidental burn injuries (Table 2).

**Table 2:** Univariate and multivariate logistic regression analysis for independent predictors of mortality among patients with acute burn injury from Jan 2011–2022 in southern west Iran.

Variable	Univariate		Multivariate	
	Crude OR (95% CI)	*P* value	Adjusted OR (95% CI)	*P* value
Time period (2018–2022 vs. 2011–2018)	0.741 (0.659–0.832)	** < 0.001**	0.814 (0.664–0.999)	**0.048**
Gender (female vs. male)	0.503 (0.447–0.566)	** < 0.001**	0.551 (0.447–0.678)	** < 0.001**
Older age	1.023 (1.021–1.026)	** < 0.001**	1.036 (1.031–1.042)	** < 0.001**
Longer length of hospitalization	0.965 (0.958–0.972)	** < 0.001**	0.950 (0.942–0.958)	** < 0.001**
Higher total body surface area	1.109 (1.103–1.115)	** < 0.001**	1.114 (1.107–1.121)	** < 0.001**
Burn etiology scald	11.589 (8.509–15.784)	** < 0.001**	1.081 (0.722–1.620)	0.705
Burn etiology contact	13.905 (5.165–37.431)	** < 0.001**	0.631 (0.212–1.883)	0.409
Burn etiology fire and flame	0.274 (0.243–0.309)	** < 0.001**	0.502 (0.399–0.633)	** < 0.001**
Burn etiology explosion or blast	–	0.999	–	–
Burn etiology electrical	6.506 (3.727–11.354)	** < 0.001**	0.841 (0.409–1.726)	0.636
Burn etiology chemical	7.151 (2.260–22.625)	**0.001**	0.723 (0.211–2.475)	0.605
Burn intention accidental	10.097 (8.537–11.943)	** < 0.001**	2.446 (1.220–4.904)	**0.012**
Burn intention suicidal	0.082 (0.068–0.098)	** < 0.001**	1.379 (0.659–2.889)	0.394
Burn intention homicidal	0.452 (0.298–0.684)	** < 0.001**	–	–

Significant values are in bold.

We also evaluated our data based on univariate and multivariate analysis, to access factors correlated with the study intervals. As demonstrated in Table 3, compared to the first interval (2011–2018), the second interval of our study (2018–2022) was significantly correlated with a more female patients, higher age, lower TBSA, less burn injuries due to scald, contact, but more frequent fire and flame injuries, and also lower mortality rate (Table 3).

**Table 3:** Univariate and multivariate logistic regression analysis regarding the independent changes among variables among patients with acute burn injury between 2011 to 2018 and 2018 to 2022 in southern west Iran.

Variable	Univariate		Multivariate	
	Crude OR (95% CI)	*P* value	Adjusted OR (95% CI)	*P* value
Gender (female vs. male)	1.254 (1.139–1.381)	** < 0.001**	1.175 (1.061–1.301)	**0.002**
Older age	1.007 (1.005–1.009)	** < 0.001**	1.010 (1.007–1.013)	** < 0.001**
Longer length of hospitalization	0.992 (0.988–0.996)	** < 0.001**	0.996 (0.992–1.000)	0.066
Higher total body surface area	0.989 (0.987–0.991)	** < 0.001**	0.990 (0.987–0.993)	** < 0.001**
Burn etiology scald	0.845 (0.756–0.944)	**0.003**	0.867 (0.757–0.995)	**0.042**
Burn etiology contact	0.248 (0.177–0.348)	** < 0.001**	0.323 (0.228–0.459)	** < 0.001**
Burn etiology fire and flame	1.499 (1.360–1.652)	** < 0.001**	1.217 (1.085–1.365)	**0.001**
Burn etiology explosion or blast	–	0.999	–	–
Burn etiology electrical	0.900 (0.726–1.115)	0.335	–	–
Burn etiology chemical	0.700 (0.459–1.068)	0.098	–	–
Burn intention accidental	0.463 (0.394–0.544)	** < 0.001**	0.881 (0.589–1.318)	0.538
Burn intention suicidal	2.350 (1.973–2.799)	** < 0.001**	1.468 (0.950–2.269)	0.084
Burn intention homicidal	1.230 (0.832–1.818)	0.300	–	–
Mortality	1.350 (1.202–1.517)	** < 0.001**	0.673 (0.557–0.814)	** < 0.001**

Significant values are in bold.

**Figure 4:**
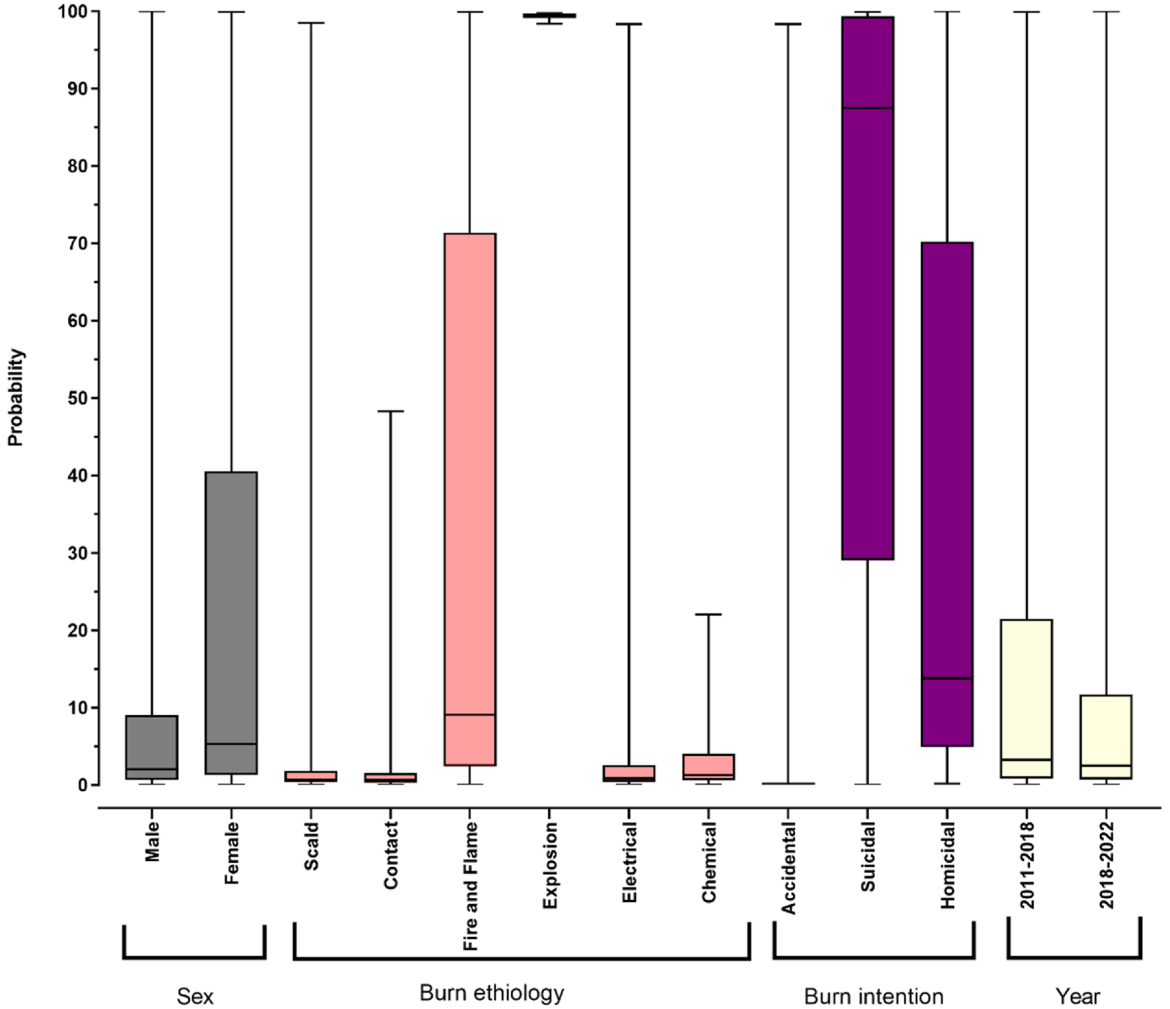
Box and wiscker plot showing mortality probability. Results (median, IQR) are showen for subgroups defined by sex (gray), burn ethiology (pink), burn intention (purple), and year (yellow).

After adjusting variables based on cofounding factors explored in the multiple regression analysis, the overall average probability of death in our study was 18.71%. The mortality probability for the study intervals were 20.67% (SD 33.0%) for 2011–2018, and 17.02% (SD 29.9%) for 2018–2022 (*P *< 0.001). Figure 4 demonstrates the probability of mortality among the variables in our study.

The LA50 was 52.15 ± 2 for all patients. This ammount was 50 ± 2% in 2011–2018, and 54 ± 2 in 2018–2022 (*P *< 0.001).

**Figure 5:**
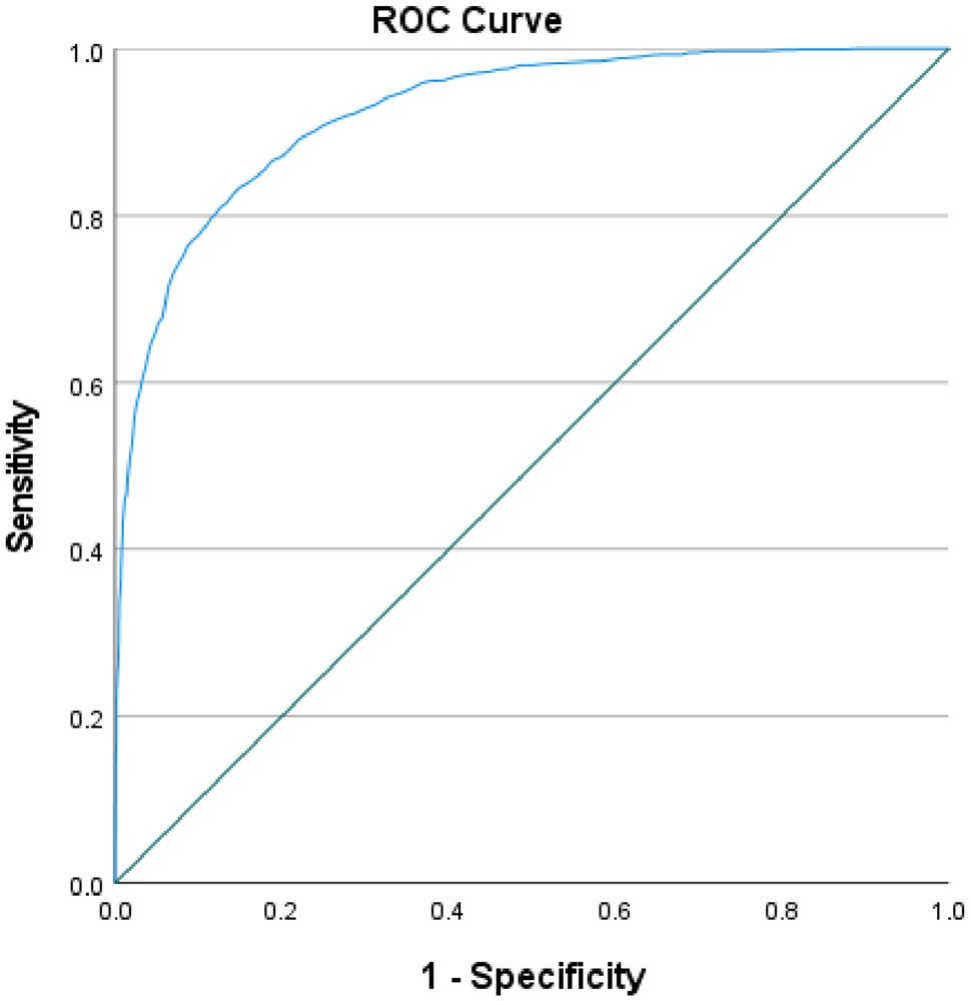
Receiver operating characteristic curve analysis of Baux score based on mortality among hospitalized patients due to acute burn injuries.

We also calculated the Baux score in our study, in which ranged from 2 to 186 [Median 53; IQR 49]. The Baux score was significantly higher during 2018–2022 compared to 2011–2018 (*P *< 0.001), and also significantly higher in patients who passed away (*P *< 0.001). Furthermore, the Baux score had a significant direct correlation with probability of mortality in our study (Correlation coefficient 0.901; *P *< 0.001). Based on ROC curve analysis, with an AUC of 0.926, the Baux score demonstrated a satisfactory correlation with mortality. Based on Fig. 5, a Baux score of 76.5 had a sensitivity of 81.1%, specificity of 87.3%, accuracy of 86.1%, positive predictive value of 60.1%, negative predictive value of 95.1% in predicting mortality among our patients.

## Discussion

This study was primarily designed to investigate patient’s outcomes between two time intervals of 2011–2018 and 2018–2021, when significant modifications have been made to our approach toward management of critically ill burn victims. These modifications include rapid transfer of severe burn patients to the ICU, establishing a well-trained multidisciplinary team, improved infection control, and utilization of specialized equipment for the management of burn patients. Our findings showed that the overall mortality was significantly decreased in patients after these modifications. In addition, this study showed a significant increase in LA50 value between the two time frames, which is an indicator of improved healthcare to the burn patients.

Generally, the LA50 value is an efficient indicator for the quality of healthcare given, particularly for patients with more severe burns^13^. It’s considered a useful standard for comparing critical care quality among burn centers or across different time periods. When we compare our results to the reports of developed countries, our results were considerably lower in terms of LA50 value. For instance, Tung et al.^18^ reported an approximate LA50 value of 80% TBSA for their patients with a mortality rate of 3.1%, which compared to our study was significantly lower^18^. A significant contributing factor to this difference could be that their study population had a mean burn area of 14% TBSA, which was considerably lower than our study where it was 24%. As our study findings suggested that TBSA is a major risk factor for higher mortality in burn patients.

Jeevan et al.^19^ found results similar to ours, with a mean LA50 of 60% TBSA estimated for burn patients in the UK. They also observed that age is a significant risk factor for increased mortality, as evidenced by LA50 values of 71.08% for patients aged 15–44, 56.64% for those aged 45–64, and 28.82% for those aged 65 and^19^. Our results showed similar findings that age is a significant factor contributing to higher mortality and lower LA50 values. Similar results were observe in the study by Roberts et al.^17^, showing lower LA50 values in advanced ages.

Reports from the developing countries showed lower rates of survival following burn injuries^20,21^, which points to the significance of critical care managements of burn victims. However, the same correlation between age and LA50 was observed. For instance, the LA50 for adults in a report from Kuwait was 76.5%, whereas for the elderly, it was 41.8%^20^. To improve outcomes for burn patients in developing countries, it appears that significant modifications in critical management, as well as the implementation of more educational and preventive programs, may be necessary. As shown by our results, implementing these changes can lead to significant improvements in patient outcomes. Aside from the changes in the LA50 value, significant changes in the Baux index across the two time intervals demonstrated similar trend in terms of patient’s outcomes. In contrast with recent literature, our findings suggested that male sex is a risk factor for unfavorable outcomes in burn patients. The results of both univariate and multivariate regression analyses in our study revealed that men are more likely to have lower LA50 values in both time intervals, compare with women. Aside from the LA50 values, the overall rate of mortality was significantly higher in men (male/female 52.6% vs. 47.4%). Also, our results indicated that the there was no difference regarding the duration of hospital stay between the two genders, which was in contrast with previous publications.

Numerous studies have examined gender differences and the influence of gender on outcomes after burn injuries. Many of these reports have shown that women have a lower proportion of injuries, smaller burn extent, and a higher incidence of scald burns compared to men, and are at a greater risk for unfavorable outcomes with extensive burn injuries, particularly with TBSA of more than 50%^22–24^. Christofides et al.^25^ reported a greater risk (OR 2.17) of mortality among female patients in South Africa. Research on burn populations in Australia and New Zealand similarly identified a higher female mortality rate (OR 2.35)^26^. There are uncertainties regarding the exact reason behind this trend. It has been thought that it can be due to the higher rate of self-immolation in female patients, which generally is associate with less favorable outcomes^27,28^. However, we speculate that the reason behind our discrepancy with the existing literature may be the difference in the overall incidence of burn injuries between the two genders. In our cohort, burn incidence was significantly higher amongst men (65.7% in men vs. 34.3% in women), which may have led to this finding. Also, men may be susceptible to traumatic events due to their higher levels of occupational exposure to injuries and comorbid diseases, which can increase their vulnerability to poor outcomes^29,30^. The same trend seems to hold true for burn injuries as well.

In line with previous reports, age was another major predictor of mortality in our study. During the two time intervals in our study, older age remained a significant non-modifiable risk factor for poor outcomes and mortality. Elderly population, most commonly referenced as ages ≥ 65 years old, are at greatest risk of death as a result of burn injuries. This elevated risk can primarily be attributed to pre-existing health conditions, age-related immunodeficiencies and skin thinning, all of which contribute to a greater extent of TBSA affected by burns after injury^31,32^.

The cause of the burn was identified as another risk factor for burn patients. Our findings suggest that injuries caused by fire and flames are more strongly associated with unfavorable outcomes in burn victims. Belba et al.^33^ reported similar findings to our study, showing that burn patients with fire as the cause of injury were 2.6 times more likely to experience mortality. When controlling for patient’s outcomes, there was no significant association between intentional injuries and mortality. Self-immolation is a devastating action and method of suicide with high fatality rates, and has been reported in our country^34,35^. Although univariant analysis reported suicide as a risk factor for unfavorable outcomes in our study cohort, the multivariable model showed no correlation between the two. Nevertheless, our study showed that accidental burns are more strongly correlated with poor outcomes, which may be attributed to the severity of burn injuries in accidental settings.

There were several limitations to this study, the primary one being that our center served as a referral center for burn injuries. Consequently, patients with minor burns may have received outpatient treatment elsewhere and were therefore not included in the study, potentially leading to underestimation of burn incidence and selection bias. However, these patients are at lower risk for mortality, and we believe that this selection does not affect the overall findings of our study regarding the LA50 values. Additionally, the study's retrospective design is another limitation. Nevertheless, this study provides one of the most comprehensive datasets on patients with burn injuries and is one of the few studies that utilized LA50 to evaluate patient outcomes.

## Conclusion

Our study suggested that higher TBSA, male gender, older age, shorter hospitalization duration, etiology of fire and flame, and accidental burn injuries are major risk factors for poor outcomes in burn victims. Also, when comparing the trend and changes in factors among burn patients after our applied policies on 2018, more female patients, higher age, lower TBSA, less burn injuries due to scald, contact, but more frequent fire and flame injuries, and also lower mortality rate was documented. In addition, the mean LA50 values showed significant improvements following significant modifications in our critical care for burn victims, including augmented intensive care unit capacity, prompt relocation of inhalation burn cases to the intensive care unit, establishing a well-trained multidisciplinary team, and improved infection control. To improve outcomes for burn patients in developing countries, major changes should be made in the management of burn patients and LA50 is a reliable assessment tool for evaluating the how these changes affect patient’s outcomes.

## Data Availability

The datasets used and/or analyzed during the current study are available from the corresponding author on reasonable request and with permission of the Research Ethics Committee of the School of Medicine-Shiraz University of Medical Sciences.
